# Determination of some phenolic compounds in *Crocus sativus* L. corms and its antioxidant activities study

**DOI:** 10.4103/0973-1296.75906

**Published:** 2011

**Authors:** N Esmaeili, H Ebrahimzadeh, K Abdi, S Safarian

**Affiliations:** *Department of Plant Biology, School of Biology, College of Science, University of Tehran, Tehran, Iran*; 1 *Department of Medicinal Chemistry and Pharmaceutical Sciences Research Center, Faculty of Pharmacy, Tehran University of Medical Sciences, Tehran, Iran*; 2 *Department of Cell and Molecular Biology, School of Biology, College of Science, University of Tehran, Tehran, Iran*

**Keywords:** Antioxidant activity, *Crocus sativus* L, GC-MS, phenolic compounds

## Abstract

It is well known that phenolic compounds are constituents of many plants. In this study, the total phenolics content in *Crocus sativus* L. corms in dormancy and waking stages were determined by the Folin-Ciocalteu method. Analysis was carried out by gas chromatography-mass spectrometry (GC-MS) after silylation by N-methyl-N-trimethylsilyl trifluroacetamide (MSTFA) + %1 trimethyl iodosilane (TMIS). Numerous compounds were detected and 11 compounds were identified. The highest phenolics content in waking corms was observed for gentisic acid (5.693 ± 0.057 μg/g) and the lowest for gallic acid (0.416 ± 0.006 μg/g); also these two phenolic compounds are the highest (0.929 ± 0.015 μg/g) and lowest (0.017 ± 0.001 μg/g) phenolics in dormant corms, respectively. The results from quantization and GC-MS analysis showed a high concentration of phenolic compounds in waking corms than the dormant stage. Furthermore, the radical scavenging activities of saffron corms were studied by 1,1-diphenyl-2-pycrylhydrazyl (DPPH) test and EC _50_values were determined about 2055 ppm and 8274 ppm for waking and dormant corms, respectively.

## INTRODUCTION

*Crocus sativus* L., commonly known as saffron, is a perennial stemless herb of the *Iridaceae* family, widely cultivated in Iran and other countries, such as Spain and Greece.[1] *C. sativus* cultivated since ancient times as the source of saffron, is a triploid plant and sterile geophyte propagated by replacement corms during period of dormancy, has recently been the subject of new studies in chemistry.[2,3]

Phenolics is a group of compounds naturally present in plants.[4] Plant-derived polyphenols receive considerable interest because of their antioxidant and antimicrobial properties. The plant polyphenols are a diverse group of higher secondary metabolites, possessing an aromatic ring bearing one or more hydroxyl substituents, derived from the shikimate pathway and phenylpropanoid metabolism. They mainly include simple phenols, phenolic acids, coumarins, tannins, and flavonoids. These compounds usually occur in the form of glycosides or esters in plants, which is the reason for their tendency to be highly water soluble.[5] Phenolic compounds have been associated with the health benefits derived from consuming high amount of fruits and vegetables.[6] The beneficial effects derived from phenolic compounds have been attributed to their antioxidant activity.[7]

Little has been published on the chemical composition or biochemistry of *C. sativus* corms. The aim of this work was to carry out a phytochemical analysis of saffron corms extracts. Our analytical attention was focused on secondary metabolites, particularly phenolic compounds from saffron corms, which have never before been reported in the literatures. These molecules play several roles in plant physiological processes, such as protection from UV, defense against pathogens, phytophagous, dormancy and allelopathic interactions. Gas chromatography-mass spectrometry (GC-MS) was selected as the method of chemical analysis to identify and quantify the metabolites in saffron corms extracts. An analytical comparison was even carried out between dormant and waking corms of *C. sativus*. Thin-layer chromatography (TLC) is still the basic tool for the separation and identification of natural compounds given in various pharmacopoeias. It is often used to provide the first characteristic fingerprints of herbal drugs that seem to be multi-component mixtures of different types of phytoconstituents.[8]

Radical scavenging activity is considered to be involved in aging processes, antiinflammatory, anticancer, and wound healing activity. The development of antioxidants that scavenge reactive oxygen species (ROS) would support biological resistance to free radicals, retard the process of aging, and decrease the risk of age-associated degenerative diseases.[9]

## EXPERIMENTAL

### Chemical and reagents

Phenolic compounds such as catechol, vanillin, *p*-hydroxybenzoic acid, cinnamic acid, 2,5-dihydroxybenzoic acid, syringic acid, gallic acid, *p*-coumaric acid, t-ferulic acid, salicylic acid, caffeic acid, and 1,1-diphenyl-2-pycrylhydrazyl (DPPH) were purchased from Sigma, USA. Folin-ciocalteu, butylated hydroxytoluene (BHT), and 2-naphthol as internal standard were purchased from Merck, Germany. All solvents from various suppliers were analytical grade and silylation reagents N-methyl-N-trimethylsilyl trifluoroacetamide (MSTFA), dithioerythritol (DTE), and ammonium iodide (NH_4_I) were obtained from Merck. The derivatizing reagent (MSTFA+1%TMIS) was prepared from MSTFA, NH_4_I, and DTE.[10]

### Instrumentation

For this research we used some instruments such as Spectrophotometer, Shimadzu UV-160; Rotary, Heidolph LABOROTA; Centrifuge Beckman J2-21M and GC-MS instrument, Trace GC Thermoquest 2000, coupled with Trace MS Thero Finigan.

### Plant material

Saffron corms were obtained from the University of Tehran farm located in Karaj, near Tehran. Dormant corms have been collected in summer and wakings in the end of winter. These corms unearthed, depleted from their sheating leaves, and cleaned from any dirt particles were kept in −70°C refrigerator.

### Sample preparation and derivatization

The extraction method used for samples was as follows: 210 ml of methanol (80%) containing BHT (1 mg/ml) were added to 30 g fresh sample and well mixed for 30 min, and 30 ml of 6M HCl were added. The mixture was stirred for 20 min, was sonicated for 5 min, and then refluxed in water bath at 90°C for 2 h and finally filtered. The supernatant was washed (three times) with *n*-hexane to eliminate small amounts of triglycerides and discarded.[4] The aqueous phase was then extracted with 60 ml (3 × 20 ml) of ethyl acetate and organic layer was separated. Anhydrous Na_2_SO_4_ was added and filtered because of the high sensitivity of trimethylsilyl (TMS) derivatives toward moisture and the residue was dried under reduced pressure.[5] One milliliter of 2-naphtol as internal standard solution (2 ppm) was added to each sample. The mixture was dried under reduced pressure and taken under vacuum oven for 24 h. For the silylation procedure, 200 μl of mixture of MSTFA+1% TMIS was added to known amount of QC sample and sonicated in water bath 70°C for 30 min.[10,11] One micro liter of silylated sample was injected to a GC-MS system.

### GC-MS condition

The GC-MS system consisted of a Thermoquest trace GC2000 coupled with a Thermo finnigan trace MS in the EI^+^ (Electron Impact) mode with electron energy set at 70 ev and the mass range at m/z (50–800). A capillary column DB-5 (30 m × 0.25 mm, i.d.) with a 0.25 μm film thickness of coated material was used. The injector was set at 270°C and transfer line temperature was set at 270°C. GC was performed in the splitless mode with 0.5 min splitless time. The temperature program was as follows: from 140°C hold for 2 min to 270°C with 5°C min^−1^, hold for 20 min. The flow rate of carrier gas (helium) was maintained at 1 ml min^−1^. Identification of compounds was achieved by compounds and the spectral data obtained from the Wiley registry of mass spectral data 7^th^ edition Xcalibur format libraries. Each determination was carried out in triplicate.

### Standard solutions and calibration curves

All analyzed compounds were prepared at 30 μg/ml concentration levels. Immediately before calibration serial dilutions with appropriate solvents were made for all the stock solutions to give eight different concentration levels for each standard. The obtained dilutions were directly injected in triplicate into the GC-MS system. The linearity was plotted using linear regression of standard area / internal standard area vs. concentration. Statistical analysis parameters were shown in Table 1. For each peaks, where signal/noise >3 accepted as limit of detection (LOD) and signal/noise >10 for limit of quantification (LOQ). LOD for all standard phenolic compounds was 1.5 ng/ml.

**Table 1:** Statistical analysis parameters of 11 standard phenolic compounds

Analyte	Calibration equation (linear model)	Correlation Factor^a^, R^2^	Precision^b^
Catechol	y = 0.4653x + 0.1709	R^2^ = 0.998	5.19
Vanillin	y = 0.4592x + 0.2171	R^2^ = 0.9952	8.24
Salicylic acid	y = 0.4594x + 0.1582	R^2^ = 0.9954	7.98
Cinnamic acid	y = 0.4871x + 0.5766	R^2^ = 0.9549	8.56
*p*-hydroxybenzoic acid	y = 0.4775x + 0.3096	R^2^ = 0.9881	3.07
Gentisic acid	y = 0.4566x + 0.2199	R^2^ = 0.9963	2.94
Syringic acid	y = 0.4511x + 0.2699	R^2^ = 0.9932	6.02
*p*- Coumaric acid	y = 0.469x + 0.2028	R^2^ = 0.9956	4.1
Gallic acid	y = 0.4459x + 0.6263	R^2^ = 0.9301	2.5
*t*- Ferulic acid	y = 0.4831x + 0.1076	R^2^ = 0.9969	5.82
Caffeic acid	y = 0.462x + 0.3018	R^2^ = 0.9886	4.99

a correlation coefficient for 8 data points in the calibration curves (*n* = 3)

b RSD% on 1 μg/ml standard anlysis (*n* = 5)

### Assay of total phenols

There are several methods to measure the content of total phenols in plant materials. The Folin-Ciocalteu[12] assay is a modification of Folin-Denis reagent as it produces less interfering precipitates, which can be problematic if samples are high in K^+^ ions.[13] A 10 g of fresh corms were homogenated by 50 ml ethanol 50%, ethanol 80%, methanol 80%, acetone 80% and water, with three replications for each sample and centrifuged for 45 min at 10.000 rpm and supernatants were collected. A suitable aliquot of the solution under test is diluted with water to about 7 ml in a 10-ml test tube. The content is well mixed with 0.5 ml of the Folin-Ciocalteu reagent. After three minutes, 1 ml of saturated sodium carbonate solution was added and the mixture made up to 10 ml. After 45 min the absorption is determined in 760 nm by spectrophotometer.[14]

### Statistical analysis

Each experiment was repeated at least three times. Analysis of variance was conducted using one-way *ANOVA* test using *SPSS* 14 for *Microsoft Windows* and means were compared by Duncan tests at the 0.05 level of confidence.

### Determination of phenolic groups by thin layer chromatography (TLC)

TLC is a relatively cheap but powerful technique to screen plant extracts for the presence of different types of phenolic compounds. Extracts were spotted on to layers of silica gel 60GF_254_ that is attached to glass sheets. All TLC plates were activated at 130°C for 1 h before use. Typical solvents are butan-2-ol /acetic acid/water (14:1:5, upper phase, v/v).[13]

The relative position or R_f_ values of the phenolic spots can be used as a first indication of phenolic compounds. Under UV-light, galloyl esters and gallo tannins appear as violet fluorescent, which are enhanced on fumigation with ammonia vapor. Plates can be sprayed with various reagents to detect phenolics compounds. The FeCl_3_-K_3_Fe(CN)_6_ spray reveals all phenolics as blue spots. Rhodamine B spray used for polyphenols and flavonols by pink spots. Saturated KIO_3_ reveals gallic acid and galloyl esters by brown or orange-brown spots. We also used NaO_2_/6% acetic acid for elagic acid by brown spots. One percent AlCl_3_ solution in ethanol by yellow fluorescence reveals flavonoids and blue spots reveal phenolic acids.

### DPPH radical scavenging test

#### Sample preparation

Ten gram of dormant and waking saffron corms were extracted by methanol 80% for 72 h and centrifuged at 7000 rpm for 30 min. The supernatant was collected and evaporated under reduced pressures.

#### DPPH test

The potential antioxidant activity of plant extracts was determined on the basis of the scavenging activity of the stable DPPH free radical.[15] The antiradical capacity of the samples was estimated according to the procedure reported by Brand-Williams[16] and slightly modified. Thirty milligrams of dried sample of dormant and waking corms resolved in 5 ml methanol stock and the solutions with various concentrations prepared. The 0.2 mM of DPPH solution in methanol used as a stock of DPPH, for determination of free radical scavenging activity of samples, 1.4 ml of each sample mixed with 0.6 ml of DPPH solution, after 30 min in room temperature, the solution absorption was measured at 515 nm by the Shimadzu UV-160 spectrophotometer versus methanol as a blank. The antiradical activity was calculated by the following ratio: (A_control_- A_sample_/ A_control_) × 100, where A_control_ is the absorption of the DPPH solution and A_sample_ is the absorption of the DPPH solution after the addition of the sample.[17]

## RESULTS AND DISCUSSION

### Total content of phenolic compounds

Table 2 shows the total content of phenolic compounds extracted by various solvents. The results have shown that using ethanol 50% in waking corms and water in dormant is more effective in phenolics extraction. Determination of phenolics analysis has shown higher content in waking corms than dormants. The increasing of phenolics content at the end of waking stage is the main reason for inducing dormancy in saffron corms and protects them against various stresses.

**Table 2:** Total phenolics content in *Crocus sativus* L. corms by Folin-Ciocalteu assay

Various solvents	Total phenolics content (mg/g) fresh weight corms			
	Waking corms		Dormant corms	
	Mean ± SD	RSD%	Mean ± SD	RSD%
Water	5.933 ± 0.033	0.556	2.220 ± 0.019	0.855
Acetone 80%	4.230 ± 0.058	1.371	2.059 ± 0.003	0.145
Ethanol 80%	4.983 ± 0.019	0.381	1.985 ± 0.036	1.813
Methanol 80%	4.599 ± 0.030	0.652	1.317 ± 0.023	1.746
Ethanol 50%	6.646 ± 0.020	0.3	1.705 ± 0.004	0.234

Results are mean ± SD (*n* = 3)

### Detection of the phenolics groups by TLC

Under short UV-light four violet spots with R_f_ values 0.18, 0.57, 0.66, and 0.88, were detected such that their intensities were enhanced on fumigation with ammonia vapor. Using rhodamine B, four pink spots emerged in dormant samples with R_f_ values 0.24, 0.59, 0.7, and 0.85, three pink spots in waking samples with R_f_ values 0.2, 0.5, and 0.85. Only one spot with R_f_ value 0.62 in waking samples was detected when saturated KIO_3_ solution was used. We also used other reagents but we could not detect further phenolics groups.

By this method, we can suggest the presence of some phenolics groups such as galloyl esters, gallo tannins, gallic acid, polyphenols, and flavonols. We could demonstrate some differences between waking and dormant samples by TLC method; for example gallic acid was detected just in waking sample, probably because of its low concentration in dormant sample, that we proved this suggestion by GC-MS analysis.

### GC-MS analysis

**Figure 1:**
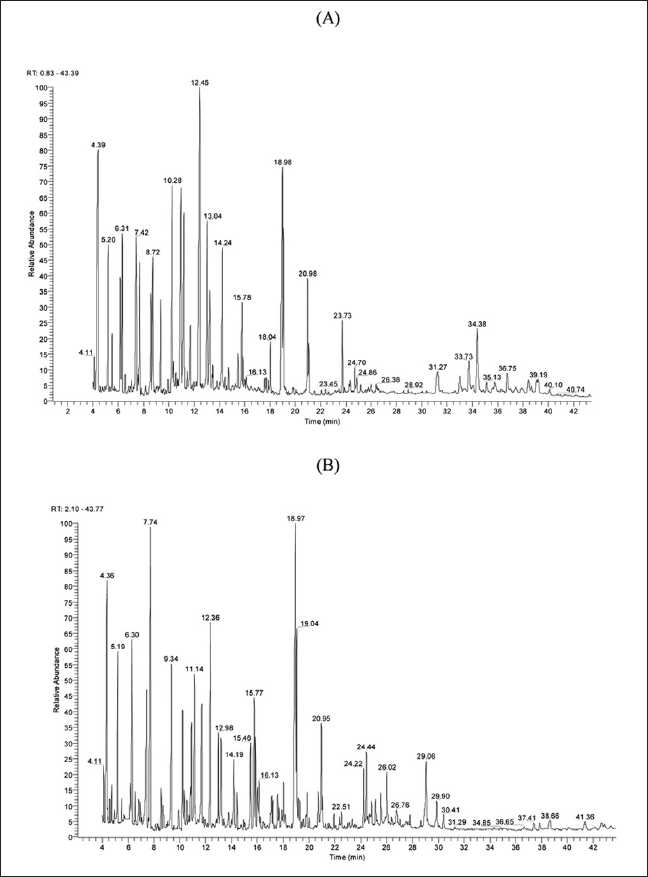
TIC chromatograms of phenolic compounds (TMS derivatives) of *Crocus sativus* L. dormant corms (A), waking corms (B)

**Figure 2:**
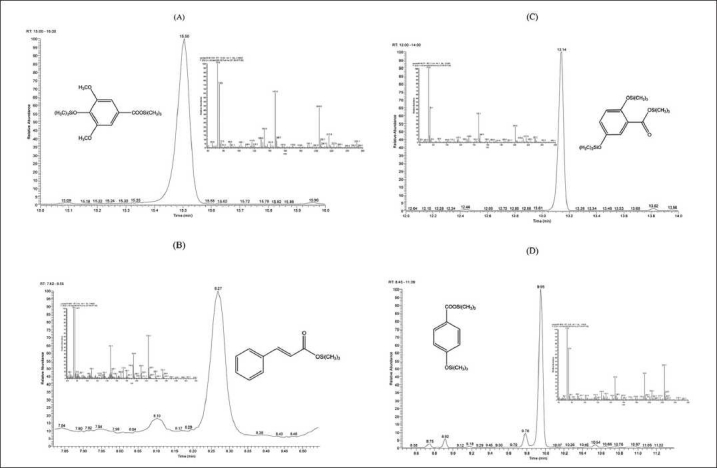
(A) SIM chromatogram of syringic acid, which was obtained from TIC chromatogram (B) SIM chromatogram of cinnamic acid, which was obtained from TIC chromatogram (C) SIM chromatogram of gentisic acid, which was obtained from TIC chromatogram (D) SIM chromatogram of *p*-hydroxybenzoic acid, which was obtained from TIC chromatogram

Before the employing GC-MS for the determination of phenolic compounds in plant extracts, a standard mixture of all substances was analyzed, after derivatization. In our samples, several free phenols were detected.Figure 1 shows the total ions chromatogram (TIC) obtained from samples of dormant (A) and waking (B), *C. sativus* L. corms. The selective ion monitoring (SIM) chromatograms also obtained from TIC chromatogram according to molecular fragment and typical retention time of analyzed standards, for example, syringic acid-2TMS, cinnamic acid-TMS, gentesic acid-3TMS, *p*-hydroxybenzoic acid-2TMS, SIM chromatograms, and shown in Figure 2A–D.

As shown in Table 1, the method was linear over the range 5-3000 ng/ml; the R^2^ values of the calibration curves were in all cases >0.9 (n = 3). LOD for all standard phenolic compounds was 1.5 ng/ml also precision value (%RSD) on 1 μg/ml standard analysis (n = 5) were determined. The results were obtained for linearity, correlation factor, and precision were satisfaction.

Retention times of silylated phenolic compounds in examined plant extract are presented in Table 3. Their molecular weights (MW) and characteristic fragments are presented also in Table 3. For example, caffeic acid-TMS showed a molecular ion (M^+^) at m/z 396 and a main peak at m/z 219. The fragmentation mechanism of simple phenols, such as caffeic acid-TMS has already been studied by other researchers. Other phenolic compounds such as catechol, vanillin, salicylic acid, cinnamic acid, *p*-hydroxybenzoic acid, 2,5-dihydroxybenzoic acid (gentisic acid), syringic acid, *p*-coumaric acid, gallic acid, and t-ferulic acid, were identified by the present method using MSTFA+1%TMIS, based upon the Wiley and NIST libraries. The concentration of phenolics was determined by comparison between the area under curve of these compounds and 2-naphtol concentration as internal standard and phenolics standards concentration. Table 4 shows the concentration of phenolics in dormant and waking corms of saffron. The highest phenolic content in waking corms was observed for gentisic acid (5.693 ± 0.057 μg/g) and the lowest for gallic acid (0.416 ± 0.006 μg/g), also these two phenolic compounds are the highest (0.929 ± 0.015 μg/g) and lowest (0.017±0.001 μg/g) phenolics in dormant corms, respectively. We obtained sufficient results to demonstrate that the concentration of phenolic compounds of saffron corms is higher in waking than dormancy stage. This experiment proved that silyl derivatization offers a very good alternative for the identification of phenolic compounds. However, it should be stressed that more research is needed on identification of silyl derivatives. The GC-MS analysis results prove the spectrophotometric data of total phenolics assay.

**Table 3:** Phenolic compounds detected in Saffron corms, retention time, molecular weight, characteristic fragments

Phenolic compounds	Retention time (RT) min	Molecular weight of TMS derivatives	Characteristic fragments
Catechol	4.41	254	73, 254, 239
Vanillin	8.15	224	194, 209, 73, 224
Salicylic acid	7.71	282	267,73, 45, 135
Cinnamic acid	8.27	220	205, 161, 131, 103, 220
*p*-hydroxybenzoic acid	9.94	282	267,193,223,282
Gentisic acid	13.14	370	355,281,147,223,267,370
Syringic acid	15.5	342	327, 342, 312, 267, 297
*p*- Coumaric acid	16.12	308	219,293,308,249
Gallic acid	16.77	458	281,458,443,355,399,179
*t*- Ferulic acid	19.07	338	338,308,323,249,293,219
Caffeic acid	19.87	396	219,396,381,191

**Table 4:** The content of phenolic compounds detected in saffron corms (μg/g) and comparison between waking and dormant corms

Phenolic compounds	Dormant (μg/g) Mean ±SD RSD%	Waking(μg/g) Mean ± SD RSD%	Waking/Dormant
Catechol	0.108 ± 0.091 8.486	2.085 ± 0.103 4.94	17.73
Vanillin	0.447 ± 0.044 9.851	1.106 ± 0.092 8.396	2.48
Salicylic acid	0.183 ± 0.005 3.095	2.845 ± 0.079 4.932	16.13
Cinnamic acid	0.03 ± 0.001 5.035	0.512 ± 0.033 6.551	17.09
*p*-hydroxybenzoic acid	0.104 ± 0.005 5.598	1.294 ± 0.091 7.044	12.41
Gentisic acid	0.929 ± 0.015 1.628	5.693 ± 0.057 4.437	6.14
Syringic acid	0.144 ± 0.012 8.701	2.084 ± 0.071 6.385	14.65
*p*- Coumaric acid	0.324 ± 0.007 2.225	4.816 ± 0.019 4.04	14.84
Gallic acid	0.017 ± 0.001 3.33	0.416 ± 0.006 3.076	24.35
*t*- Ferulic acid	0.114 ± 0.009 8.288	2.254 ± 0.076 3.414	19.37
Caffeic acid	0.289 ± 0.017 6.108	14.125 ± 0.045 1.512	47.88

Results are mean ± SD (*n* = 3)

### DPPH test

**Figure 3:**
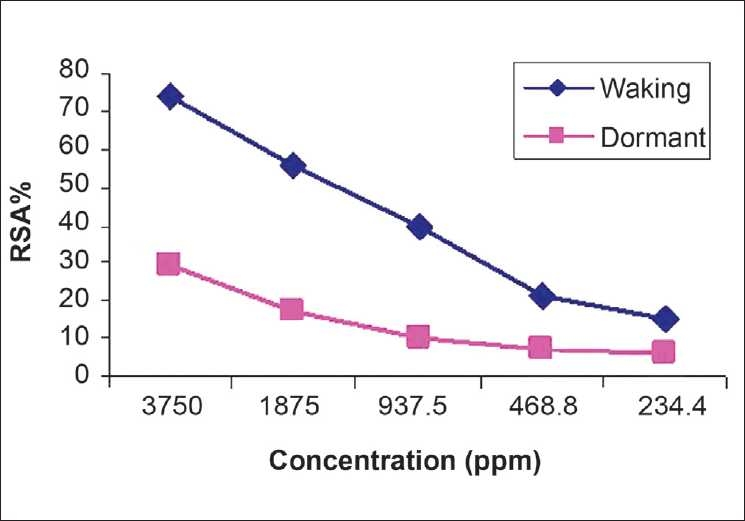
Antiradical activity of waking and dormant corms of saffron

The presence of phenolic compounds in saffron corms is an important result for the biological property (antioxidants and antiradicals) of these metabolites; thus, for their possible application in various industrial activities, such as foof/feed, cosmetic, and phytomedicine. Moreover, further developments could be previewed about the evaluation of antimicrobial and antimycotic effects of these secondary metabolites present in *C. sativus* L. corms in order to test the possible biomedical interests. In the present study the possible radical scavenging activity of saffron corms was examined. The radical scavenging activity of saffron corms presented in Figure 3. As shown in Table 5, the methanol extract of *C. sativus* L. corms at a concentration in 3750 ppm, exhibited significant radical scavenging activity (%RSA) of about (74 ± 0.763) in waking and (29 ± 0.854) in dormant corms. The EC_50_ values were determined for each sample for waking and dormant corms and it was 2055 ppm and 8274 ppm, respectively. However, the results show higher antioxidant activity in waking corms than the dormant, in the same concentration of extract [Table 5]. The EC_50_ values also confirmed this conclusion that the antioxidant activity in waking stage is higher than dormancy. The significant antioxidant activity of waking corms of *C. sativus* methanol extract should probably be attributed to a synergistic action of main bioactive constituents such as phenolic compounds.[18]

**Table 5:** Radical scavenging activities of *Crocus sativus* L. corms

Concentration (ppm)	%RSA (Waking)	%RSA (Dormant)
234.375	14.8 ± 1.101	6.4 ± 0.45
468.75	21.1 ± 0.709	7 ± 0.55
937.5	39.3 ± 1.171	10 ± 0.611
1875	55.6 ± 0.611	17 ± 0.655
3750	74 ± 0.763	29 ± 0.854

Results are mean ± SD (*n* = 3)
